# *Ganoderma lucidum* and *Robinia pseudoacacia* Flower Extract Complex Alleviates Kidney Inflammation and Fibrosis by Modulating Oxidative Stress

**DOI:** 10.3390/antiox14040409

**Published:** 2025-03-28

**Authors:** Soyoung Kim, Jeongwon Kim, Jong-Lae Kim, Mi-Ryeong Park, Kye Won Park, Ki Wung Chung

**Affiliations:** 1 HLscience Co., Ltd., Uiwang-si 16004, Republic of Korea; sykim@hlscience.com (S.K.); kimjl@hlscience.com (J.-L.K.); alfud1030@hlscience.com (M.-R.P.); 2 Department of Food Science and Biotechnology, Sungkyunkwan University, Suwon 16419, Republic of Korea; kwpark@skku.edu; 3 Department of Pharmacy and Research Institute for Drug Development, College of Pharmacy, Pusan National University, Busan 46241, Republic of Korea; 98juon_k@naver.com

**Keywords:** *Ganoderma lucidum*, *Robinia pseudoacacia*, NEPROBIN, CKD, inflammation

## Abstract

Chronic kidney disease (CKD) is characterized by functional and structural abnormalities, with its progression strongly influenced by oxidative stress and inflammatory responses, ultimately leading to renal fibrosis. This study aimed to investigate the effects of a *Ganoderma lucidum* and *Robinia pseudoacacia* flower extract complex (NEPROBIN) through in vitro and in vivo experiments. In vitro experiments with NRK52E renal tubular epithelial cells demonstrated that NEPROBIN significantly alleviates H_2_O_2_-induced oxidative stress and suppresses lipopolysaccharide (LPS)-induced activation of the mitogen-activated protein kinase (MAPK) and nuclear factor kappa-light-chain-enhancer of activated B cells (NF-κB) signaling pathways. Additionally, NEPROBIN reduced LPS-induced NF-κB transcriptional activity and downregulated the expression of cytokines and chemokines in these cells. We further investigated the effects of NEPROBIN in vivo. Kidney damage was induced in mice using a 0.25% adenine diet (AD), and the mice were orally treated with NEPROBIN at doses of 100, 200, and 400 mg/kg/day for two weeks. NEPROBIN treatment significantly reduced AD-induced elevations in blood urea, serum creatinine, and urinary β2-microglobulin levels. Markers of oxidative stress and kidney damage were notably lower in the kidneys of NEPROBIN-treated mice. Furthermore, NEPROBIN effectively mitigated the AD-induced inflammatory response in the kidneys, with a marked reduction in cytokine and chemokine expression. This decrease in inflammation was associated with a significant reduction in tubulointerstitial fibrosis. Overall, NEPROBIN alleviated renal damage and fibrosis by directly targeting renal oxidative stress and inflammation, highlighting its potential as a therapeutic agent for CKD.

## 1. Introduction

Chronic kidney disease (CKD) is a significant global health concern, affecting millions of individuals worldwide and contributing to high morbidity and mortality rates [1]. It is characterized by persistent functional and structural abnormalities of the kidneys, which impair their ability to maintain homeostasis in the body. These abnormalities can manifest as decreased glomerular filtration rate (GFR), proteinuria, or structural damage detectable via imaging or biopsy. CKD often progresses to end-stage renal disease, a life-threatening condition that requires renal replacement therapy, such as dialysis or kidney transplantation. The development of kidney fibrosis is a key pathological process in CKD [2]. Understanding the mechanisms underlying renal fibrosis is crucial for developing therapies that can prevent or slow the progression of CKD. In response to tissue injury, wound-healing mechanisms are activated to control inflammation and promote tissue regeneration. However, persistent inflammation hinders complete regeneration, leading to the formation of fibrotic scar tissue [3]. The excessive deposition of extracellular matrix during chronic and pathological fibrosis disrupts the kidney’s normal architecture, impairing its function. Ultimately, unresolved kidney fibrosis progresses to an irreversible stage, contributing to renal failure.

Oxidative stress and inflammation are central to the pathogenesis of CKD, playing a crucial role in exacerbating renal damage and fibrosis [4]. Oxidative stress occurs when there is an imbalance between the production of reactive oxygen species (ROS) and the body’s ability to neutralize them with antioxidants [5]. In the kidneys, ROS can damage cellular structures, including lipids, proteins, and DNA, triggering a cascade of inflammatory responses [6]. This oxidative damage directly contributes to tubular injury, endothelial dysfunction, and fibrosis. Multiple cell types, including myeloid-derived inflammatory cells, fibroblasts, pericytes, epithelial cells, and endothelial cells, contribute to the inflammatory response. Recent studies have increasingly focused on the role of tubular epithelial cells (TECs) in renal fibrosis and inflammation [7]. The sustained activation of pro-inflammatory signaling pathways like nuclear factor kappa-light-chain-enhancer of activated B cells (NF-κB) and mitogen-activated protein kinase (MAPK) in TECs further amplifies the inflammatory response, promoting the progression of renal fibrosis and exacerbating kidney damage [8]. Given their central role in CKD pathogenesis, targeting oxidative stress and inflammation presents a promising therapeutic strategy to halt or even reverse the progression of CKD.

*Ganoderma lucidum,* commonly known as Reishi or Lingzhi, is a prominent medicinal mushroom belonging to the Ganodermataceae family [9]. Renowned for its therapeutic potential, *G. lucidum* has been utilized in traditional Asian medicine for over two millennia, particularly in Chinese, Japanese, and Korean cultures, where it is highly regarded for its purported health benefits, including immune modulation, anti-inflammatory effects, and anti-cancer properties [10]. The bioactive compounds present in *G. lucidum*, such as triterpenoids, polysaccharides, and peptides, have garnered significant scientific attention due to their diverse pharmacological activities [11]. *Robinia pseudoacacia*, or black locust, is a deciduous tree native to North America and distributed globally in temperate regions [12]. The tree produces fragrant white or yellow flowers, which have been traditionally used in herbal medicine to treat various ailments, including gastrointestinal and skin disorders [13]. *R. pseudoacacia* contains various bioactive compounds, including flavonoids, phenolic compounds, alkaloids, tannins, essential oils, and polysaccharides, which contribute to its antioxidant, anti-inflammatory, antimicrobial, and hepatoprotective properties [13]. Although the medicinal properties of *G. lucidum* and *R. pseudoacacia* have been explored, their potential role in the development of CKD remains under-investigated. This study aims to evaluate the effects of a *G. lucidum* and *R. pseudoacacia* flower extract complex on kidney disease using both in vitro and in vivo models.

## 2. Materials and Methods

### 2.1. Preparation of NEPROBIN Complex

The complex (NEPROBIN) of reishi (*Ganoderma lucidum*) fruiting body extracts powder (RFE) and false acacia (*Robinia pseudoacacia* L.) flower extracts powder (FFE) was provided by HL Science Co., Ltd. (Uiwang, Republic of Korea) and composed of RFE:FFE = 1:1. RFE was obtained by extracting the Reishi fruiting body with 50% ethanol, concentrating, and drying. Additionally, FFE was obtained by extracting the false acacia flower with 50% ethanol, concentrating, and drying. NEPROBIN contained 0.80 mg/g ± 20% Ganoderic acid A and 10.00 mg/g ± 20% Robinin. NEPROBIN was kept at −20 °C until the experiments were performed.

### 2.2. High-Performance Liquid Chromatography (HPLC) Analysis

The HPLC system (Waters corporation, Milford, MA, USA), which consisted of the Waters e2695 system equipped with a 2998 PDA detector and Empower 3 software, was used. Ganoderic acid A was performed using Capcellpak C18 UG120 column, 4.6 × 250 mm, 5 μm. The analyte was eluted 2% acetic acid in water (*v*/*v*) and acetonitrile with stepwise elution mode. Column temperature was set at 25 °C and the flow rate was 1.0 mL/min. The detection wavelength was set at 254 nm. Robinin was performed using Capcellpak C18 MG column, 4.6 × 250 mm, 5 μm. The analyte was eluted 0.1% acetic acid in water (*v*/*v*) and acetonitrile with stepwise elution mode. Column temperature was set at 25 °C and the flow rate was 1.0 mL/min. The detection wavelength was set at 280 nm.

### 2.3. Animal Experiments

All animal experiments were performed in accordance with the guidelines for animal experimentation issued by Pusan National University (PNU) and were approved by the Institutional Animal Care Committee of PNU (IACUC approval No. PNU-2024-0412). The male C57BL/6J mice were purchased from Hyochang Science (Daegu, Republic of Korea). All mice were maintained at 23 ± 2 °C with a relative humidity of 60 ± 5% and 12 h light/dark cycles and were provided with free access to water and food. The animals were randomly sorted into four different groups (*n* = 9~10). To induce CKD, mice (9–10 weeks old) were fed a 0.25% adenine diet (AD) (JA BIO, Suwon, Republic of Korea) for two weeks. To evaluate the potential renoprotective effects of natural compounds, NEPROBIN was administered orally at doses of 100, 200, and 400 mg/kg in 10% DMSO in water once daily. As a positive control, dapagliflozin (HY-10450, MedChemExpress, Monmouth Junction, NJ, USA) was administered orally at 10 mg/kg using the same vehicle. The control group and AD group received only 10% dimethyl sulfoxide in water as a vehicle. After the respective starvation periods, the mice were euthanized via CO_2_ inhalation and sacrificed for further analysis. Serum and kidney tissue samples were subsequently collected for biochemical analyses.

### 2.4. Determination of Nephrotoxicity

Blood creatinine and blood urea nitrogen (BUN) levels are known to accumulate in the bloodstream when kidney disease impairs renal excretion, making them useful markers of kidney function. In this study, commercially available assay kits from Shinyang Diagnostics (Seoul, Republic of Korea) were used, following the manufacturer’s protocol, to measure BUN (1120171, SICDIA L-BUN) and creatinine (1120051, SICDIA CREA) levels in mouse serum. The BUN assay is based on a urease–glutamate dehydrogenase (GLDH) enzymatic method, where urease hydrolyzes urea to ammonia, and GLDH catalyzes the conversion of ammonia to glutamate with a concomitant reduction of NADH, leading to a measurable decrease in absorbance [14]. The creatinine assay employs the Jaffe reaction, which quantifies creatinine based on its reaction with picric acid in an alkaline medium, forming a colorimetric complex [15]. β2-Microglobulin is a sensitive biomarker of kidney toxicity, as its elevated levels in urine indicate impaired tubular reabsorption and early renal dysfunction. To analyze urinary β2-microglobulin, mouse urine was collected using a metabolic cage on the last day of the experiment. Urinary β2-microglobulin levels were measured using the Mouse Beta-2-Microglobulin enzyme-linked immunosorbent assay kit (Abcam, Cambridge, UK, ab223590). The assay was performed using the antibodies provided in the kit, and absorbance was measured at 450 nm using a spectrophotometer (Berthold Technologies GmbH & Co., Bad Wildbad, Germany). The concentration of urinary β2-microglobulin was calculated based on the standard curve included in the kit. Curve validation was assessed by determining the linearity (R^2^ value > 0.99), ensuring a strong correlation between concentration and absorbance.

### 2.5. Cell Culture Experiments and Cytotoxicity Assessment of NEPROBIN

The rat tubular epithelial cell line (NRK-52E) was obtained from ATCC and cultured in Dulbecco’s Modified Eagle’s Medium (DMEM) supplemented with 5% fetal bovine serum (FBS) and 1% penicillin. Cells were maintained at 37 °C in a humidified incubator with 95% air and 5% CO_2_. NEPROBIN was prepared as a 20 mg/mL stock solution in DMSO and stored at −20 °C until use. Cell viability following treatment with natural compounds was assessed using the MTT (3-(4,5-Dimethylthiazol-2-yl)-2,5-diphenyltetrazolium bromide) assay with the EZ-Cytox Cell Viability Assay Kit (Dogen, Seoul, South Korea), following the manufacturer’s instructions. NRK-52E cells were cultured in 96-well plates until reaching 70–80% confluence. They were then treated with various concentrations of NEPROBIN in serum-free media for 24 h. All wells, except for blanks, received 1% DMSO. After incubation, the EZ-Cytox reagent was added, and the plates were incubated for 30 min. Absorbance was measured using a spectrophotometer, and cell viability was calculated as follows: Cell viability (%) = (Absorbance of blank group / Absorbance of treated group) × 100.

### 2.6. Determination of Antioxidant Effect

ROS generation was assessed using the fluorescent dye 2′,7′-dichlorodihydrofluorescein diacetate (DCFDA) in both in vitro and in vivo models. For the in vitro model, NRK52E cells were pretreated with NEPROBIN or N-acetyl cysteine (NAC) for 30 min, followed by stimulation with 300 µM of hydrogen peroxide (H_2_O_2_) for 1 h. After treatment, cells were harvested and centrifuged to remove the supernatant. The cell pellet was then resuspended in 50 μM DCFDA dissolved in PBS and incubated at 37 °C for 10 min. After incubation, the cells were centrifuged at 1000× *g* for 10 min again to remove the supernatant and resuspended in PBS. The samples were then transferred to a 96-well black plate, and fluorescence intensity was measured using a GENios plate reader (Tecan Instruments, Salzburg, Austria) with an excitation wavelength of 485 nm and an emission wavelength of 530 nm. For the in vivo model, kidney tissue was homogenized, and the homogenized sample was incubated with 50 μM DCFDA dissolved in PBS at 37 °C for 10 min. Fluorescence intensity was measured using the same protocol as the in vitro model.

To assess intracellular glutathione (GSH) levels, a GSH assay was performed in ean in vitro model. Prepared cell pellets were homogenized and treated with 25% meta-phosphoric acid, followed by centrifugation to collect the supernatant. The supernatant was mixed with 1 mM EDTA in 50 mM phosphate buffer, followed by the addition of o-phthalaldehyde (P0657, Sigma-Aldrich, St. Louis, MO, USA). After incubation at room temperature for 20 min, fluorescence intensity was measured using an excitation wavelength of 360 nm and an emission wavelength of 460 nm. GSH concentrations were quantified using a GSH standard curve, ensuring accuracy and linearity. For both ROS and GSH assays, fluorescence intensity values were normalized to the protein concentration of each sample.

The EZ-Lipid Peroxidation (TBARS) Assay Kit from Dogenbio (Seoul, Republic of Korea) was used to measure malondialdehyde (MDA) levels in renal tissue. The TBARS assay is a well-known method for screening lipid peroxidation by measuring the MDA-TBA adduct, which is formed when MDA reacts with thiobarbituric acid (TBA). Tissue samples were homogenized in PBS containing 1X BHT, and the supernatant was used for the experiment. After adding the acid reagent and indicator solution, 150 µL of the mixture was dispensed into a microplate, and absorbance was measured at 540 nm using a plate reader. To accurately determine the MDA concentration, a standard curve was generated using MDA samples of known concentrations, and validated for precision and reproducibility. Curve validation was assessed by determining the linearity (R^2^ value > 0.99), ensuring a strong correlation between concentration and absorbance.

### 2.7. Total RNA Extraction and qPCR

Total RNA extraction was performed using RiboEx™ reagent (GeneAll Biotechnology Co., Ltd., Seoul, Republic of Korea) following the manufacturer’s guidelines. Briefly, tissue and cell samples were homogenized in 500 µL of RiboEx™ reagent, followed by mixing with 100 µL of chloroform. After centrifugation, the aqueous phase was collected and precipitated with isopropyl alcohol to obtain the RNA pellet. The pellet was washed with 75% ethanol, air-dried, and resuspended in diethyl pyrocarbonate (DEPC)-treated water. RNA concentration and purity were assessed using a NanoDrop spectrophotometer (NanoDrop 2000, Thermo Fisher Scientific, Waltham, MA, USA). For cDNA synthesis, 0.1 µg of total RNA was reverse-transcribed using SuPrimeScript RT Premix (GENETBIO Inc., Daejeon, Republic of Korea) according to the manufacturer’s protocol. Quantitative real-time polymerase chain reaction (qPCR) was performed using the synthesized cDNA, Prime Q-Master Mix (GENETBIO Inc., Daejeon, Republic of Korea), DEPC water, and specific primers. The specific primers were designed using Primer3Plus (Table 1). qPCR was conducted using the CFX Connect System (Bio-Rad, Hercules, CA, USA). For data analysis, the 2^−ΔΔCT^ method was applied for relative quantification.

### 2.8. Protein Extraction and Western Blotting

For in vitro protein extraction, harvested NRK52E cells were lysed by adding Cell Lysis Buffer (10X) (Cat# 9803, Cell Signaling Technology, Inc., Danvers, MA, USA) and Xpert Protease Inhibitor Cocktail Solution (100X) (Cat# P3100-001, GenDEPOT, Barker, TX, USA) in PBS. For in vivo protein extraction, homogenized tissue samples were processed using ProEXTM CETi (Translab, Daejeon, Republic of Korea) according to the manufacturer’s instructions. The protein concentration of extracted samples was determined using the Pierce™ Dilution-Free™ Rapid Gold BCA Protein Assay (Cat# A55861, Thermo Fisher Scientific), and absorbance was measured at 562 nm using a spectrophotometer. For Western blotting, extracted proteins were prepared by mixing with 4× sample buffer (Cat# 1610747, Bio-Rad, CA, USA) prior to electrophoresis. Western blotting was performed using the Bio-Rad Western blot system. Briefly, 10–20 µg of protein was separated by SDS-PAGE and transferred onto polyvinylidene difluoride (PVDF) membranes (Millipore, Burlington, MA, USA). The membranes were then blocked for 1–2 h using blocking buffer containing 5% non-fat milk or 3% BSA in 0.1% Tween-Tris buffer (10 mM Tris, pH 7.5, 100 mM NaCl, and 0.1% Tween 20). Following blocking, the membranes were incubated overnight at 4 °C with specific primary antibodies diluted at 1:500 to 1:2000 in Tris buffer (10 mM Tris, pH 7.5, 100 mM NaCl) (Table 2). The next day, the membranes were incubated with horseradish peroxidase (HRP)-conjugated anti-mouse or anti-rabbit secondary antibodies (1:10,000 dilution) at 25 °C for 1 h. Protein bands were visualized using the Western Bright Peroxide solution (Advansta, San Jose, CA, USA) and detected using a ChemiDoc imaging system (Bio-Rad, Hercules, CA, USA).

### 2.9. Determination of Transcriptional Activity

To assess transcriptional activity, a luciferase assay was performed. NRK52E cells were seeded in a 96-well plate and cultured until they reached 70–80% confluence. The cells were then transfected with 0.05 µg of NF-κB plasmid using Lipofectamine 3000 (Thermo Fisher Scientific, Waltham, MA, USA) according to the manufacturer’s instructions. After 24 h of transfection, the cells were pretreated with NEPROBIN or NAC for 2 h, followed by stimulation with 10 μg of LPS for 4 h. Luciferase activity was measured using the One-Glo Luciferase Assay System (Promega, Madison, WI, USA) and luminescence was detected using a GENios plate reader (Tecan, Männedorf, Switzerland).

### 2.10. Histological Analysis

Paraffin-embedded kidney sections were processed using standard protocols and stained with hematoxylin and eosin (H&E). Sirius red staining was performed on paraffin-embedded sections using the VitroView™ Picro-Sirius Red Stain Kit (VitroVivo Biotech, Rockville, MD, USA) according to the manufacturer’s instructions. Microscopic images were acquired using an LS30 microscope (Leam Solution, Seoul, Republic of Korea). The percentage of Sirius red-positive areas was quantified using ImageJ software (Version 1.54d, National Institutes of Health, Bethesda, MD, USA).

### 2.11. Quantification and Statistical Analysis

Graph generation and statistical analysis were performed using GraphPad Prism 5 software (La Jolla, CA, USA). For cell experiments, each experiment was repeated at least three times. A parametric Student’s *t*-test was used to analyze differences between the two groups. All data are presented as mean ± SEM, and a *p*-value of less than 0.05 was considered statistically significant.

## 3. Results

### 3.1. HPLC Analysis of RFE, FFE, and NEPROBIN

**Figure 1:**
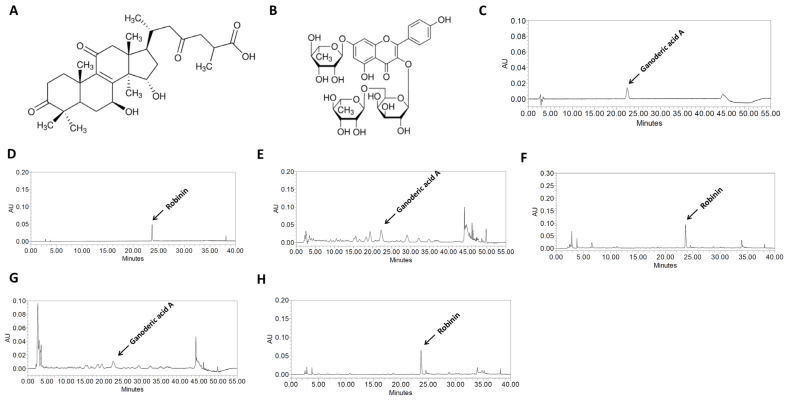
HPLC chromatograms of Ganoderic acid A and Robinin in the RFE, FFE, and NEPROBIN at 254 or 280 nm. (**A**) Ganoderic acid A and (**B**) Robinin structure, (**C**) Ganoderic acid A and (**D**) Robinin standard chromatogram, (**E**) RFE and (**F**) FFE chromatogram, (**G**) Neprobin chromatogram for Ganoderic acid A and (**H**) Robinin.

Ganoderic acid A and Robinin are representative compounds for RFE and FFE, serving as key markers for their chemical composition (Figure 1A,B). The HPLC analysis of RFE and FFE revealed two peaks matching those of the certain standards Ganoderic acid A and Robinin with retention times of approximately 22 and 23 min, respectively (Figure 1C,D). The RFE and FFE contained 1.6 mg/g ± 20% Ganoderic acid A and 20 mg/g ± 20% Robinin, respectively (Figure 1E,F). The NEPROBIN also revealed two peaks matching those of the certain standards Ganoderic acid A and Robinin with retention times of approximately 22 and 23 min, respectively (Figure 1G,H). Furthermore, the NEPROBIN contained 0.8 mg/g ± 20% Ganoderic acid A and 10 mg/g ± 20% Robinin, respectively.

### 3.2. G. lucidum and R. pseudoacacia Flower Extract Complex (NEPROBIN) Attenuate H_2_O_2_-Induced Oxidative Stress and Lipopolysaccharide-Induced Inflammation in NRK52E Cells

**Figure 2:**
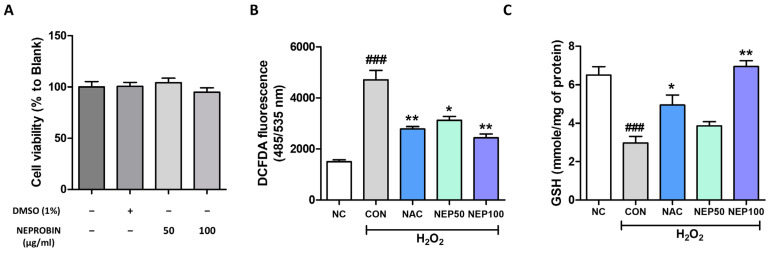
NEPROBIN treatment alleviates oxidative stress in NRK52E renal tubule epithelial cells. (**A**) Cytotoxicity was measured after treatment with different doses of NEPROBIN. (**B**) Oxidative stress was assessed using the DCFDA (2′,7′-Dichlorofluorescin diacetate) fluorescent dye in NRK52E cells treated with H_2_O_2_ and/or NEPROBIN. N-acetyl cysteine (NAC) treatment served as a positive control. ### *p* < 0.001 vs. the untreated control group; ** *p* < 0.01, * *p* < 0.05 vs. the H_2_O_2_-treated group. (**C**) Cellular glutathione (GSH) levels were measured in NRK52E cells treated with H_2_O_2_ and/or NEPROBIN. ### *p* < 0.001 vs. the untreated control group; ** *p* < 0.01, * *p* < 0.05 vs. the H_2_O_2_-treated group.

The effects of NEPROBIN on H_2_O_2_-induced oxidative stress in NRK52E renal tubular epithelial cells were examined. A cell viability assay revealed that NEPROBIN exhibited no significant toxicity at doses up to 100 μg/mL (Figure 2A). The level of oxidative stress determined by DCFDA assay was significantly higher in the H_2_O_2_-treated cells than in the non-treated cells (Figure 2B). A substantial reduction was observed in the NAC 1 mM, NEPROBIN 50 μg/mL, and NEPROBIN 100 μg/mL treatment groups, showing reductions of 41%, 33.7%, and 48.2%, respectively (Figure 2B). GSH levels were further determined. The levels of GSH were significantly lower in H_2_O_2_-treated cells compared to non-treated cells (Figure 2C). Significant improvements were observed in the NAC and NEPROBIN 100 μg/mL treatment groups, with increases of 66.8% and 134.5%, respectively (Figure 2C).

**Figure 3:**
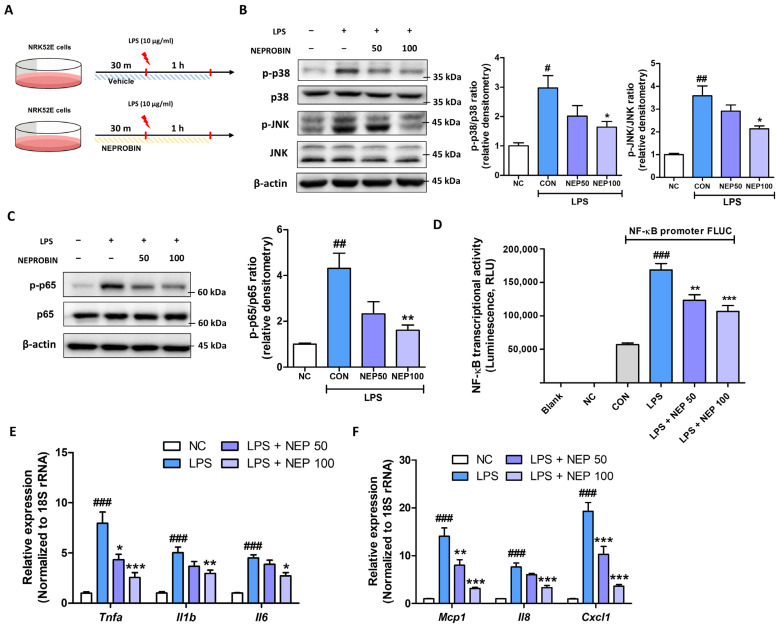
NEPROBIN treatment attenuates LPS-induced p38 and JNK activation and pro-inflammatory responses in NRK52E cells. (**A**) Experimental scheme demonstrating the anti-inflammatory effects of NEPROBIN in NRK52E cells treated with LPS. (**B**) Protein expression of p38, phosphorylated p38, JNK, and phosphorylated JNK in total lysates of NRK52E cells under LPS treatment with or without NEPROBIN. β-actin was used as the internal control. Relative protein expressions were quantified using densitometry. # *p* < 0.05 vs. the untreated control group. ## *p* < 0.005 vs. the untreated control group. * *p* < 0.05 vs. the LPS-treated group. (**C**) Protein expression of p65 and phosphorylated p65 in total lysates of NRK52E cells under LPS treatment with or without NEPROBIN. β-actin was used as the internal control. Relative protein expressions were quantified using densitometry. ## *p* < 0.01 vs. the untreated control group. ** *p* < 0.01 vs. the LPS-treated group. (**D**) NF-κB transcriptional activity was measured by luciferase assay under LPS treatment with or without NEPROBIN treatment. ### *p* < 0.001 vs. the untreated control group. ** *p* < 0.01, *** *p* < 0.001 vs. the LPS-treated group. (**E**) Relative mRNA levels of inflammation-related cytokine genes (*Tnfa, Il1b*, and *Il6*) in LPS-treated NRK52E cells with or without NEPROBIN. ### *p* < 0.001 vs. the untreated control group. * *p* < 0.05, ** *p* < 0.01, *** *p* < 0.001 vs. the LPS-treated group. (**F**) Relative mRNA levels of inflammation-related chemokine genes (*Mcp1, Il8*, and *Cxcl1*) in LPS-treated NRK52E cells with or without NEPROBIN. ### *p* < 0.001 vs. the untreated control group. ** *p* < 0.01, *** *p* < 0.001 vs. the LPS-treated group.

Next, we checked whether NEPROBIN attenuates lipopolysaccharide (LPS)-induced inflammatory responses in NRK52E cells (Figure 3A). NEPROBIN (100 μg/mL) treatment significantly reduced LPS-induced phosphorylation of p38 and JNK by 45.9% and 41.4%, respectively (Figure 3B). The effect of NEPROBIN on NF-κB activation was further examined. NEPROBIN (100 μg/mL) treatment significantly reduced LPS-induced phosphorylation of p65 by 62.8% (Figure 3C). The transcriptional activity of NF-κB was further assessed, and NEPROBIN treatment at 50 μg/mL and 100 μg/mL significantly reduced LPS-induced NF-κB transcriptional activity by 26.8% and 36.8%, respectively (Figure 3D). Consequently, the effect of NEPROBIN on LPS-induced cytokine and chemokine expression was evaluated at the gene level. NEPROBIN treatment significantly reduced LPS-induced expression of cytokine genes (*Tnfa, Il1b*, and *Il6*) and chemokine genes (*Mcp1, Il8*, and *Cxcl1*) in the cells. The reduction was most pronounced in the NEPROBIN 100 µg/mL group, with decreases of 64% for *Tnfa*, 41.2% for *Il1b*, 39.3% for *Il6*, 77.6% for *Mcp1*, 56.3% for *Il8*, and 81.2% for *Cxcl1* compared to the LPS-treated control group (Figure 3E,F). Together, these data suggest that NEPROBIN alleviates oxidative stress and reduces inflammatory responses in renal tubule epithelial cells.

### 3.3. NEPROBIN Attenuates the Decline in Renal Function in a Mouse Model Fed an Adenine Diet (AD)

**Figure 4:**
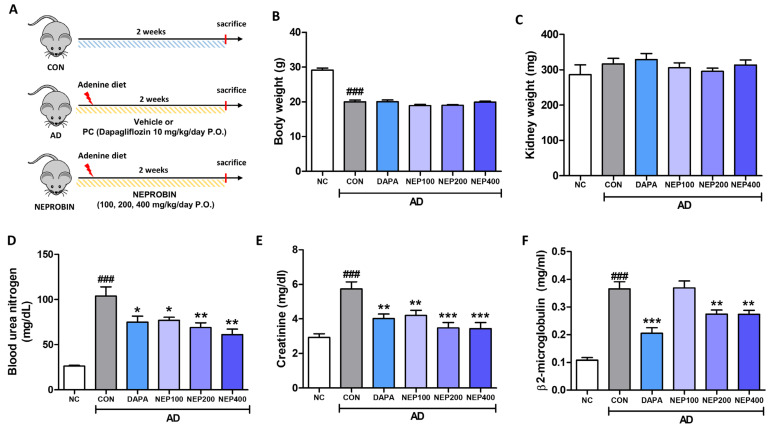
NEPROBIN reduces renal damage in adenine diet (AD)-induced kidney fibrosis model. (**A**) Scheme for animal experiments. Dapagliflozin treatment served as a positive control in animal experiments. (**B**) Body weight at the end of the experimental schedule. ### *p* < 0.001 vs. the untreated control group. (**C**) Kidney weight at the end of the experimental schedule. (**D**) Blood urea nitrogen levels were measured to evaluate the kidney function of experimental animals. ### *p* < 0.001 vs. the untreated control group. * *p* < 0.05, ** *p* < 0.01 vs. the AD-fed group. (**E**) Serum creatinine levels were measured to evaluate the kidney function of experimental animals. ### *p* < 0.001 vs. the untreated control group. ** *p* < 0.01, *** *p* < 0.001 vs. the AD-fed group. (**F**) Urinary β2-microglobin levels were measured to evaluate the kidney function of experimental animals. ### *p* < 0.001 vs. the untreated control group. ** *p* < 0.01, *** *p* < 0.001 vs. the AD-fed group.

Based on its demonstrated anti-oxidative stress and anti-inflammatory properties under in vitro experimental conditions, we further investigated the effects of NEPROBIN in a mouse model of CKD induced by a two-weeks of AD. Dapagliflozin (10 mg/kg/day) was used as a positive control, while NEPROBIN was administered orally by gavage at doses of 100, 200, and 400 mg/kg/day for two weeks (Figure 4A). Administration of AD significantly reduced body weight after two weeks, while neither dapagliflozin nor NEPROBIN influenced body weight changes throughout the experimental period (Figure 4B). No significant changes in kidney weight were observed after two weeks of AD administration (Figure 4C). We next evaluated parameters of kidney function markers in the blood and urea. BUN levels were significantly increased in the AD group compared to the control group, and a substantial reduction was observed in the dapagliflozin, NEPROBIN 100 mg, NEPROBIN 200 mg, and NEPROBIN400 mg treatment groups, showing reductions of 27.9%, 25.9%, 33.6%, and 41.2%, respectively (Figure 4D). Serum creatinine level was also elevated in the AD group, and a substantial reduction was observed in the dapagliflozin, NEPROBIN 100 mg, NEPROBIN 200 mg, and NEPROBIN 400 mg treatment groups, showing reductions of 30%, 26.8%, 39.5%, and 40.2%, respectively (Figure 4E). Urinary β2-microglobulin levels were significantly increased in the AD group compared to the control group, and a substantial reduction was observed in the dapagliflozin, NEPROBIN 200 mg, and NEPROBIN 400 mg treatment groups, showing a reduction of 44%, 24.9%, and 25.2%, respectively (Figure 4F). These results suggest that NEPROBIN significantly attenuates the AD-induced decline in renal function, as indicated by serum and urinary markers of renal function.

### 3.4. NEPROBIN Reduces Oxidative Stress and Prevents Renal Structural Damage in a Mouse Model Fed an AD

**Figure 5:**
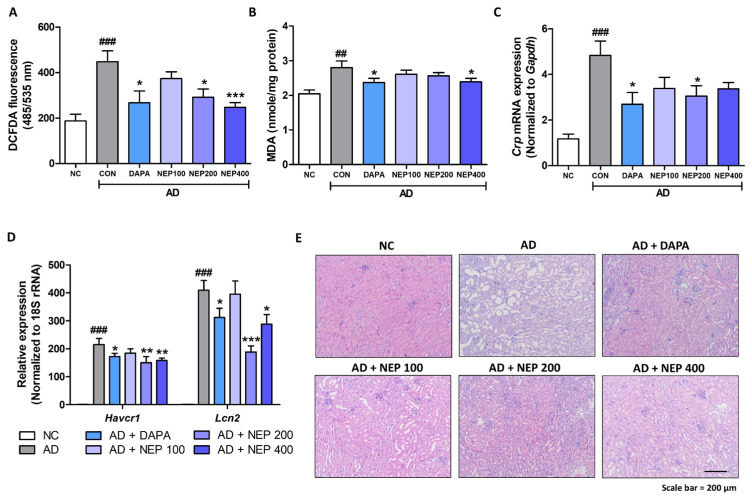
NEPROBIN alleviates oxidative stress and renal structural damage in AD-induced kidney fibrosis model. (**A**) Oxidative stress was assessed using the DCFDA fluorescent dye in kidneys. ### *p* < 0.001 vs. the untreated control group. * *p* < 0.05, *** *p* < 0.001 vs. the AD-fed group. (**B**) Malondialdehyde levels were measured to evaluate kidney oxidative stress in animal experiments. ## *p* < 0.01 vs. the untreated control group. * *p* < 0.05 vs. the AD-fed group. (**C**) mRNA expression of *Crp* gene in animal experiments. ### *p* < 0.001 vs. the untreated control group. * *p* < 0.05 vs. the AD-fed group. (**D**) Relative mRNA levels of kidney damage-related genes (*Havcr1*, and *Lcn2*) in animal experiments. ### *p* < 0.001 vs. the untreated control group. * *p* < 0.05, ** *p* < 0.01, *** *p* < 0.001 vs. the AD-fed group. (**E**) Representative H&E staining images of kidneys.

We further assessed oxidative stress and renal structural damage in the same mouse model. Oxidative stress, measured by DCFDA, was significantly increased in the kidneys of AD-fed mice. In contrast, reduced oxidative stress was observed in the dapagliflozin, NEPROBIN 200 mg, and NEPROBIN 400 mg treatment groups, showing reductions of 40%, 34.9%, and 45%, respectively (Figure 5A). The levels of MDA, another marker of oxidative stress, were also evaluated. MDA levels were significantly increased in the kidneys of AD-fed mice, while the dapagliflozin and NEPROBIN 400 mg treatment groups exhibited reduced MDA levels, with decreases of 15.5% and 14.8%, respectively (Figure 5B). The gene expression of Crp, which is known to be elevated in kidney disease conditions, was evaluated. AD-fed kidney showed increased Crp expression, while the dapagliflozin and NEPROBIN 200 mg treatment groups exhibited reduced expression, with decreases of 44.3% and 37.1%, respectively (Figure 5C). The gene expression of kidney damage markers (*Havcr1* and *Lcn2*) was significantly reduced in the dapagliflozin, NEPROBIN 200 mg, and NEPROBIN 400 mg treatment groups, particularly in the NEPROBIN 200 mg group (30.2% for *Havcr1*, and 54% for *Lcn2* compared to the AD-treated control group) (Figure 5D). Structural changes were further assessed using H&E staining. The kidneys of AD-fed mice exhibited significant alterations, including tubule dilation, tubular atrophy, and infiltration of inflammatory cells. The dapagliflozin and NEPROBIN-treated groups exhibited fewer structural changes, suggesting a reduction in renal damage induced by AD (Figure 5E). Collectively, these data suggest that NEPROBIN effectively reduces oxidative stress and prevents renal structural damage in a mouse model fed an AD.

### 3.5. NEPROBIN Suppresses the MAPK and NF-κB Signaling Pathways in a Mouse Model Fed an AD, Resulting in Reduced Cytokine and Chemokine Expression in the Kidney

**Figure 6:**
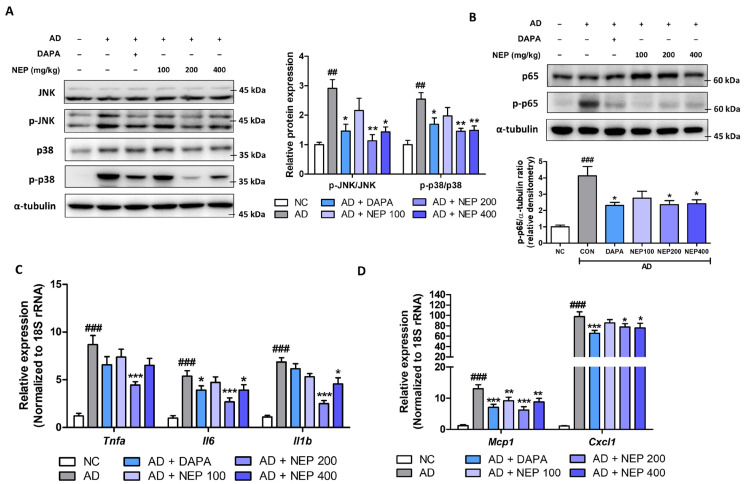
NEPROBIN modulates p38 and JNK activation and pro-inflammatory responses in AD-induced kidney fibrosis model. (**A**) Protein expression of p38, phosphorylated p38, JNK, and phosphorylated JNK in kidneys. α-tubulin was used as the internal control. Relative protein expressions were quantified using densitometry. ## *p* < 0.01 vs. the untreated control group. * *p* < 0.05, ** *p* < 0.01 vs. the AD-fed group. (**B**) Protein expression of p65 and phosphorylated p65 in kidneys. α-tubulin was used as the internal control. Relative protein expressions were quantified using densitometry. ### *p* < 0.001 vs. the untreated control group. * *p* < 0.05 vs. the AD-fed group. (**C**) Relative mRNA levels of inflammation-related cytokine genes (*Tnfa, Il6*, and *Il1b*) in kidneys. ### *p* < 0.001 vs. the untreated control group. * *p* < 0.05, *** *p* < 0.001 vs. the AD-fed group. (**D**) Relative mRNA levels of inflammation-related chemokine genes (*Mcp1,* and *Cxcl1*) in kidneys. ### *p* < 0.001 vs. the untreated control group. * *p* < 0.05, ** *p* < 0.01, *** *p* < 0.001 vs. the AD-fed group.

We further evaluated whether NEPROBIN reduces inflammatory responses in the AD kidney model. Consistent with the in vitro experiments, NEPROBIN significantly decreased the phosphorylation of JNK, p38, and p65 in the kidneys. The NEPROBIN 200 mg and 400 mg treatment groups showed a significant reduction in p-JNK levels (61% and 50.6%, respectively, compared to the AD group) and in p-p38 levels (42.8% and 41.5%, respectively, compared to the AD group) in the kidney (Figure 6A). The p-p65 levels were significantly reduced in the NEPROBIN 200 mg and 400 mg treatment groups, with decreases of 33.1% and 42.7%, respectively (Figure 6B). The reduction in MAPK and NF-κB signaling led to decreased cytokine and chemokine expression in the kidneys. The gene expression of cytokines (*Tnfa*, *Il6*, and *Il1b*) and chemokines (*Mcp1* and *Cxcl1*) was significantly lower, particularly in the NEPROBIN 200 mg (*Tnfa*: 48.7%, *Il6*: 50.0%, *Il1b*: 63.6%, *Mcp1*: 52.4%, and *Cxcl1*: 20.6%) and 400 mg treatment groups (*Tnfa*: 25.1%, *Il6*: 27.0%, *Il1b*: 33.6%, *Mcp1*: 32.3%, and *Cxcl1*: 22.5%), compared to the AD-fed control kidneys (Figure 6C,D). These findings indicate that NEPROBIN effectively suppresses MAPK and NF-κB signaling pathways in a mouse model fed an AD, leading to a significant reduction in cytokine and chemokine expression in the kidney.

### 3.6. NEPROBIN Mitigates Fibrosis Development in a Mouse Model Fed an AD

**Figure 7:**
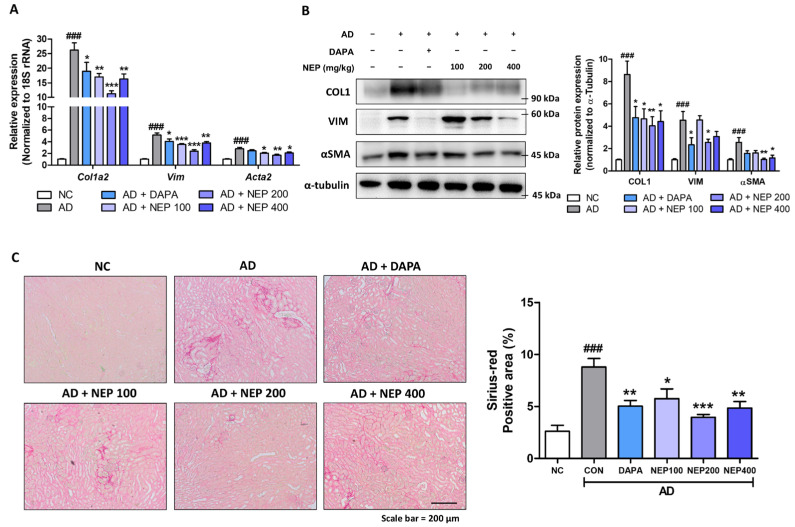
NEPROBIN reduces fibrosis response in kidneys. (**A**) Relative mRNA levels of fibrosis-related genes (*Col1a2, Vim*, and *Acta2*) in kidneys. ### *p* < 0.001 vs. the untreated control group. * *p* < 0.05, ** *p* < 0.005, *** *p* < 0.001 vs. the AD-fed group. (**B**) Protein expression of COL1, Vimentin, and α-SMA in kidneys. α-tubulin was used as the internal control. Relative protein expressions were quantified using densitometry. ### *p* < 000.1 vs. the untreated control group. * *p* < 0.05, ** *p* < 0.01 vs. the AD-fed group. (**C**) Representative images of Sirius Red staining of kidneys. Positive staining areas were quantified. ### *p* < 0.001 vs. the untreated control group. * *p* < 0.05, ** *p* < 0.01, *** *p* < 0.001 vs. the AD-fed group.

Lastly, we assessed the extent of fibrosis in the same animal model. The NEPROBIN-treated groups exhibited significantly reduced gene expression of extracellular matrix proteins (*Col1a2*, *Vim*, and *Acta2*) in the kidneys compared to the AD-fed control group (Figure 7A). The reduced gene expression was particularly detected in the NEPROBIN 200 mg (*Col1a2*: 57.1%, *Vim*: 53.7%, and *Acta2*: 39.2%) and 400 mg treatment (*Col1a2*: 37.8%, *Vim*: 26.1%, and *Acta2*: 24.6%) groups, compared to the AD-fed control kidneys. The protein expression also showed similar tendency to gene expression levels. NEPROBIN treatment significantly reduced COL1, Vimentin, and α-SMA protein expression compared to the AD-fed control group (Figure 7B). The reduced protein expression was particularly detected in the NEPROBIN 200 mg (COL1: 53.3%, Vimentin: 43.4%, and α-SMA: 59.8%) and 400 mg treatment (COL1: 48.7%, Vimentin: 32.1%, and α-SMA: 54.3%) groups, compared to the AD-fed control kidneys. The extent of fibrosis was further assessed histologically using Sirius Red (SR) staining. The SR staining revealed that NEPROBIN treatment significantly reduced fibrotic regions in the interstitial area (Figure 7C). When the positive region for SR staining was calculated, a substantial reduction was observed in the NEPROBIN 100 mg, NEPROBIN 200 mg, and NEPROBIN 400 mg treatment groups, showing reductions of 34.7%, 55%, and 44.9%, respectively (Figure 7C). Collectively, these results demonstrate that NEPROBIN effectively attenuates fibrosis development in the kidneys of the AD-fed mouse model.

## 4. Discussion

Previous studies have demonstrated the potent antioxidant properties of *G. lucidum* and *R. pseudoacacia* flower extract, highlighting their potential therapeutic applications. *G. lucidum* shows significant antioxidant activity in various in vitro and in vivo models, including hepatotoxicity, trauma-induced oxidative stress, and gamma radiation-induced damage [16]. It reduces lipid peroxidation, restores mitochondrial function, and enhances antioxidant enzyme activity, thus protecting against oxidative stress-related damage [17]. In neuronal models, *G. lucidum* mitigates oxidative stress-induced brain injury by modulating inflammatory pathways [18]. Similarly, *R. pseudoacacia* flower extract, rich in phenolic compounds like flavonols and hydroxycinnamic acid derivatives, exhibits strong free radical scavenging activity, reducing oxidative damage [19]. While both extracts show promise in various contexts, their specific effects in kidney disease and their role in mitigating oxidative stress in renal pathologies remain largely unexplored, warranting further investigation. In our study, we aimed to evaluate whether the antioxidant effects of the *G. lucidum* and *R. pseudoacacia* flower extract complex could reduce inflammatory responses under both in vitro and in vivo conditions. We assessed its impact on key inflammatory markers and oxidative stress parameters, investigating its potential as a therapeutic agent for renal protection. By examining these effects, our study provides new insights into the possible nephroprotective properties of this extract combination.

The kidney is responsible for excreting metabolic waste through urine. Waste products like urea, creatinine, and uric acid prevent toxin accumulation and uremia [20]. The European Uremic Toxin Work Group has classified over 100 uremic toxins, with urea nitrogen, creatinine, and β2-microglobulin being key examples [21,22]. These toxins activate inflammatory markers such as C-reactive protein (CRP), nitric oxide (NO), and NF-κB, increasing pro-inflammatory cytokines (IL-6, IL-1, TNF-α) and worsening inflammation [23]. Additionally, uremic toxins elevate oxidative stress by promoting ROS and oxidative damage markers like MDA, peroxynitrite, and advanced glycation end products, further driving inflammation [24]. As kidney function declines, toxin accumulation increases, exacerbating inflammation, oxidative stress, and associated complications such as cardiovascular disease [25]. Managing uremic toxin levels is crucial for maintaining kidney health. While dietary control is the primary approach, recent studies have explored prebiotics, probiotics, and synbiotics for modulating gut microbiota to reduce toxin levels [26]. Building on this, our study aims to develop functional materials derived from natural sources to improve uremic toxin excretion.

The kidneys, due to their high metabolic activity and oxygen consumption, are particularly vulnerable to oxidative damage [27]. An imbalance between ROS production and antioxidant defense mechanisms leads to oxidative stress, which contributes to renal injury. Oxidative stress is closely linked to various pathogenic processes in CKD, including inflammation, endothelial dysfunction, and fibrosis [28]. Excessive ROS levels damage essential cellular components, including lipids, proteins, and DNA, leading to apoptosis, necrosis, and impaired kidney function. Furthermore, ROS activate key inflammatory pathways, such as MAPK and NF-κB pathways, exacerbating cytokine production and immune cell infiltration in renal tissues [29]. Several studies have explored the therapeutic potential of antioxidants in CKD management. Compounds such as N-acetylcysteine (NAC), vitamin E, and polyphenols have demonstrated the ability to reduce oxidative stress and improve kidney function in experimental and clinical settings [30,31]. Additionally, natural extracts, including those derived from medicinal plants, have gained attention for their dual antioxidative and anti-inflammatory effects [32,33]. By targeting ROS-induced damage, these compounds may not only alleviate renal inflammation and fibrosis but also improve vascular health and overall kidney function. Similar to previous results that highlight the protective effects of natural antioxidants, NEPROBIN complex showed promising antioxidative and anti-inflammatory properties in both in vitro and in vivo models of kidney injury. Its ability to scavenge ROS, modulate inflammatory pathways, and preserve renal function suggests its potential as a therapeutic agent for mitigating oxidative stress-related kidney damage.

The MAPK and NF-κB signaling pathways are closely interconnected and play pivotal roles in inflammation [34]. The MAPK pathway, activated by stress signals and inflammatory stimuli regulates the production of pro-inflammatory mediators through key kinases like ERK, JNK, and p38. NF-κB, a major transcription factor in inflammation, is activated by similar stimuli and promotes the expression of cytokines, chemokines, and adhesion molecules. In kidney disease development, the activation of the MAPK and NF-κB signaling pathways is consistently observed, driving inflammatory responses that contribute to CKD progression [29,35]. Inhibiting these pathways could reduce the production of pro-inflammatory cytokines, chemokines, and other mediators that drive immune cell infiltration and tissue damage. Several pharmacological agents including natural compounds, including specific inhibitors of MAPK and NF-κB, have shown potential in preclinical models for reducing inflammation and fibrosis in kidney disease [36,37]. Understanding their crosstalk could lead to more effective treatments for kidney disease.

In our experiments, we successfully demonstrated that the NEPROBIN complex effectively reduces oxidative stress, inhibits the MAPK and NF-κB signaling pathways, and mitigates fibrosis in both in vitro and in vivo models. However, several limitations remain that need to be addressed in future studies. First, although the NEPROBIN complex showed a protective effect both under in vitro and in vivo condition, the exact compounds involved in its mechanism of action remain unidentified. Further research is needed to isolate and characterize the active components responsible for its protective effects. Second, while our study demonstrated significant reductions in oxidative stress and fibrosis, the long-term efficacy and potential side effects of NEPROBIN treatment require further investigation through extended in vivo studies. Third, the precise molecular interactions between NEPROBIN and key signaling pathways, including MAPK and NF-κB, need to be elucidated to confirm its direct regulatory effects. Future studies should also explore the optimal dosage and delivery methods to enhance the therapeutic potential of NEPROBIN. Additionally, clinical validation in human subjects will be essential to determine its translational relevance. Despite these limitations, our data underscore the therapeutic potential of NEPROBIN in alleviating renal damage and dysfunction by addressing both oxidative stress and inflammatory pathways.

## 5. Conclusions

The results of our study demonstrated the beneficial effects of the NEPROBIN complex in kidney disease development. More specifically, our investigation focused on elucidating the protective mechanisms of NEPROBIN against kidney injury, ranging from oxidative stress and inflammation to fibrotic progression. The antioxidative properties of NEPROBIN reduced the inflammatory response in kidney epithelial cells through the suppression of MAPK and NF-κB signaling pathways, which are key regulators of immune activation and tissue damage in CKD. Based on the in vitro observations, we further demonstrated the in vivo efficacy of NEPROBIN in an adenine-induced kidney fibrosis model, a well-established system that mimics the inflammatory and fibrotic characteristics of human CKD. NEPROBIN treatment significantly reduced kidney inflammation and fibrosis, as evidenced by decreased expression of pro-inflammatory cytokines and reduced deposition of extracellular matrix components. Collectively, these findings provide compelling evidence supporting NEPROBIN as a promising strategy for treating kidney fibrosis and related pathologies.

## Figures and Tables

**Table 1:** Primer sequences for qPCR.

Rat			
Gene	Forward (5′-3′)	Reverse (3′-5′)	GenBank^®^ Accession Number
*Mcp1*	ATGCAGTTAATGCCCCACTC	TTCCTTATTGGGGTCAGCAC	NM_031530.1
*Cxcl1*	AGACAGTGGCAGGGATTCAC	GGGGACACCCTTTAGCATCT	NM_030845.2
*Il8*	GAAGATAGATTGCACCGA	GATAGCCTCTCACACATTTC	XM_004833923.2
*Tnfa*	ATTGCTCTGTGAGGCGACTG	GGGGCTCTGAGGAGTAGACG	NM_012675.3
*Il1b*	AAAATGCCTCGTGCTGTCTG	CCACAGGGATTTTGTCGTTG	NM_031512.2
*Il6*	TCTCTCCGCAAGAGACTTCCA	ATACTGGTCTGTTGTGGGTGG	NM_012589.2
*18s rRNA*	ACAGCTGCTGCTTTCACCGT	TCAACCCACTTCTGATGGGCT	NR_046237.3
**Mouse**			
*Mcp1*	CCAGCAAGATGATCCCAATG	CTTCTTGGGGTCAGCACAGA	NM_011333.3
*Cxcl1*	AATGCATCCACATGCTGCTA	ATAGCCTCCTCGACCCACTT	NM_008176.3
*Tnfa*	CGTCAGCCGATTTGCTATCT	CGGACTCCGCAAAGTCTAAG	D84199.2
*Il1b*	GCCCATCCTCTGTGACTCAT	AGGCCACAGGTATTTTGTCG	NM_008361.4
*Il6*	TGGGTTCTAGCCAGCAGAGT	ACCACCAGAGACCGTTATGC	NM_031168.2
*Crp*	CGCAGCTTCAGTGTCTTCTC	AGATGTGTGTTGGAGCCTCA	NM_007768.4
*Havcr1*	CTGGAATGGCACTGTGACATCC	GCAGATGCCAACATAGAAGCCC	BC053400.1
*Lcn2*	ACTGAATGGGTGGTGAGTGT	GGGAGTGCTGGCCAAATAAG	NM_008491.2
*Col1a1*	CAGCTCCAGGAAGACCTCGA	GTAACAAGGGTGAGCCTGGC	NM_007742.4
*Vim*	CAAGCCTGACCTCACTGCTG	CACCTGTCTCCGGTACTCGT	NM_011701.4
*Acta2*	TTGTCCACCGCAAATGCTTC	AAGGCGCTGATCCACAAAAC	NM_007392.3
*Gapdh*	AAGGTCATCCCAGAGCTGAA	CTGCTTCACCACCTTCTTGA	GU214026.1

**Table 2:** Information of primary antibodies used in Western blotting.

Antibody	Company	Catalog Number
p-p38	Cell Signaling	9216S
p38	Santa Cruz	sc-81621
p-JNK	Invitrogen	44-682G
JNK	Santa Cruz	sc-7395
p-p65	Cell Signaling	3033L
p65	GeneTex	GTX107678
COL1	Santa Cruz	sc-393573
Vimentin	Cell Signaling	5741S
αSMA	Santa Cruz	sc-32251
α-tubulin	Santa Cruz	sc-5286
β-actin	Santa Cruz	sc-69879

## Data Availability

Data sharing is not applicable to this article.
